# Supplementary material for: Cooperation by necessity: condition- and density-dependent reproductive tactics of female house mice
Source: Commun Biol. 2022 Apr 12;5:348. doi: 10.1038/s42003-022-03267-2 (PMC9005510; doi:10.1038/s42003-022-03267-2)
Supplement: Supplementary file 2 — Supplementary Information [file 42003_2022_3267_MOESM2_ESM.pdf]

SI files

Population size and composition over time

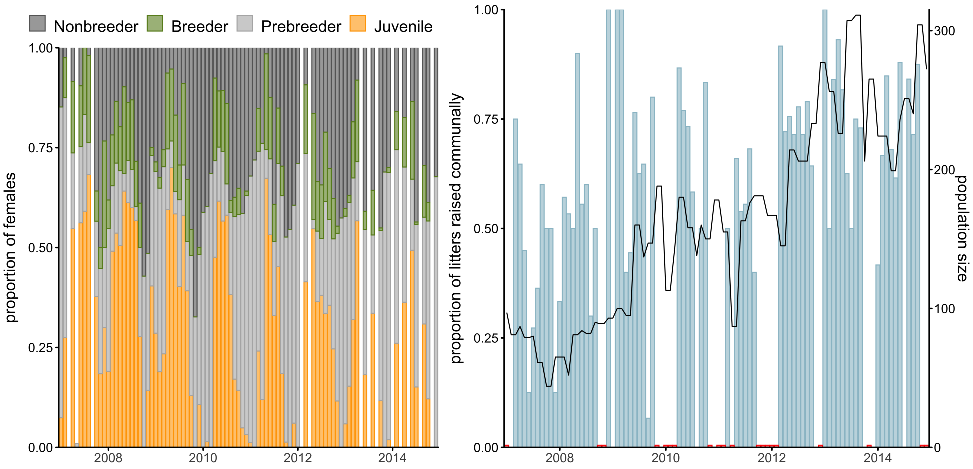

Figure S1: a) The proportion of females in each state throughout the study period (raw data). Missing values are indicated by gaps. b) The proportion of litters raised communally over time (blue bars). A red line indicates that no females were breeding in a given month. The total population size (adult males and females of at least 18g) is denoted in black.

MSCMR model selection

In a first step, we identified the best structure for detectability (p) and survival (S), while modelling transition probabilities (ψ) as a function of state:tostate (intercept removed) and its interaction with season, as needed to parameterise our matrix population model. Due to data limitations and a lack of difference between 3 of the life-history states, we further simplified the states for p and S and classified all individuals that had reproduced at least once (S, C and N) as breeding individuals, while keeping two distinct states for juveniles (J) and prebreeders (P). The Akaike information criterion, adjusted for small sample sizes (AICc) was used to select the best model (see Table S1). After having determined the best model structure, we added population size (total number of adults) as additive and interactive (with state and season) covariate to estimate p, S and ψ. AICc was again used to select the best model (see Table S2). The model with population size included both for p, S and ψ had the lowest AICc and was therefore used to parameterise the matrix population models at two different densities.

model	npar	Δ AICc	weight
S(state * season)p(state * season)ψ(-1 + (state:tostate):season)	24.00	0.00	0.99
S(state * season)p(state + season)ψ(-1 + (state:tostate):season)	22.00	10.98	0.00
S(state * season)p(state)ψ(-1 + (state:tostate):season)	22.00	11.71	0.00
S(state * season)p(state)ψ(-1 + (state:tostate):season)	21.00	42.40	0.00
S(state + season)p(state * season)ψ(-1 + (state:tostate):season)	22.00	105.01	0.00
S(state + season)p(state)ψ(-1 + (state:tostate):season)	20.00	106.82	0.00
S(state + season)p(state + season)ψ(-1 + (state:tostate):season)	20.00	113.91	0.00
S(state + season)p(state)ψ(-1 + (state:tostate):season)	19.00	142.62	0.00
S(state)p(state)ψ(-1 + (state:tostate):season)	21.00	173.80	0.00
S(state)p(state * season)ψ(-1 + (state:tostate):season)	23.00	177.87	0.00
S(state)p(state + season)ψ(-1 + (state:tostate):season)	21.00	184.47	0.00
S(state)p(state)ψ(-1 + (state:tostate):season)	20.00	208.19	0.00
S(state)p(state)ψ(-1 + (state:tostate):season)	19.00	218.63	0.00
S(state)p(state * season)ψ(-1 + (state:tostate):season)	21.00	219.34	0.00
S(state)p(state + season)ψ(-1 + (state:tostate):season)	19.00	226.39	0.00
S(state)p(state)ψ(-1 + (state:tostate):season)	18.00	249.82	0.00
S(state * season)p(state * season)ψ(-1 + (state:tostate))	20.00	401.72	0.00
S(state * season)p(state + season)ψ(-1 + (state:tostate))	18.00	415.53	0.00
S(state * season)p(state)ψ(-1 + (state:tostate))	18.00	436.38	0.00
S(state * season)p(state)ψ(-1 + (state:tostate))	17.00	449.69	0.00
S(state + season)p(state * season)ψ(-1 + (state:tostate))	18.00	508.18	0.00
S(state + season)p(state + season)ψ(-1 + (state:tostate))	16.00	519.24	0.00
S(state + season)p(state)ψ(-1 + (state:tostate))	16.00	533.81	0.00
S(state + season)p(state)ψ(-1 + (state:tostate))	15.00	550.82	0.00
S(state)p(state * season)ψ(-1 + (state:tostate))	19.00	573.19	0.00
S(state)p(state + season)ψ(-1 + (state:tostate))	17.00	581.01	0.00
S(state)p(state)ψ(-1 + (state:tostate))	17.00	591.53	0.00
S(state)p(state)ψ(-1 + (state:tostate))	16.00	607.68	0.00
S(state)p(state * season)ψ(-1 + (state:tostate))	17.00	619.66	0.00
S(state)p(state + season)ψ(-1 + (state:tostate))	15.00	628.61	0.00
S(state)p(state)ψ(-1 + (state:tostate))	15.00	639.39	0.00
S(state)p(state)ψ(-1 + (state:tostate))	14.00	654.52	0.00
S(state * season)p(season)ψ(-1 + (state:tostate):season)	20.00	1344.80	0.00
S(state * season)p(1)ψ(-1 + (state:tostate):season)	19.00	1346.47	0.00
S(state + season)p(season)ψ(-1 + (state:tostate):season)	18.00	1436.93	0.00
S(state + season)p(1)ψ(-1 + (state:tostate):season)	17.00	1437.58	0.00
S(season)p(state * season)ψ(-1 + (state:tostate):season)	20.00	1445.57	0.00
S(season)p(state + season)ψ(-1 + (state:tostate):season)	18.00	1480.34	0.00
S(state)p(season)ψ(-1 + (state:tostate):season)	19.00	1503.71	0.00
S(state)p(1)ψ(-1 + (state:tostate):season)	18.00	1506.11	0.00
S(state)p(season)ψ(-1 + (state:tostate):season)	17.00	1544.61	0.00
S(state)p(1)ψ(-1 + (state:tostate):season)	16.00	1546.88	0.00
S(season)p(state)ψ(-1 + (state:tostate):season)	18.00	1607.00	0.00
S(season)p(state)ψ(-1 + (state:tostate):season)	17.00	1634.50	0.00
S(state * season)p(season)ψ(-1 + (state:tostate))	16.00	1724.92	0.00
S(state * season)p(1)ψ(-1 + (state:tostate))	15.00	1726.73	0.00
S(season)p(state * season)ψ(-1 + (state:tostate))	16.00	1819.10	0.00
S(state + season)p(season)ψ(-1 + (state:tostate))	14.00	1825.54	0.00
S(state + season)p(1)ψ(-1 + (state:tostate))	13.00	1826.10	0.00
S(season)p(state + season)ψ(-1 + (state:tostate))	14.00	1859.64	0.00
S(state)p(season)ψ(-1 + (state:tostate))	15.00	1883.10	0.00
S(state)p(1)ψ(-1 + (state:tostate))	14.00	1884.84	0.00
S(1)p(state * season)ψ(-1 + (state:tostate):season)	19.00	1905.12	0.00
S(state)p(season)ψ(-1 + (state:tostate))	13.00	1935.00	0.00
S(state)p(1)ψ(-1 + (state:tostate))	12.00	1937.24	0.00
S(1)p(state + season)ψ(-1 + (state:tostate):season)	17.00	1940.53	0.00
S(season)p(state)ψ(-1 + (state:tostate))	14.00	2015.23	0.00
S(season)p(state)ψ(-1 + (state:tostate))	13.00	2027.22	0.00
S(1)p(state)ψ(-1 + (state:tostate):season)	17.00	2073.39	0.00
S(1)p(state)ψ(-1 + (state:tostate):season)	16.00	2094.26	0.00
S(1)p(state * season)ψ(-1 + (state:tostate))	15.00	2255.60	0.00
S(1)p(state + season)ψ(-1 + (state:tostate))	13.00	2296.15	0.00
S(1)p(state)ψ(-1 + (state:tostate))	13.00	2453.68	0.00
S(1)p(state)ψ(-1 + (state:tostate))	12.00	2462.65	0.00
S(season)p(season)ψ(-1 + (state:tostate):season)	16.00	3448.17	0.00
S(season)p(1)ψ(-1 + (state:tostate):season)	15.00	3462.30	0.00
S(season)p(season)ψ(-1 + (state:tostate))	12.00	3838.86	0.00
S(season)p(1)ψ(-1 + (state:tostate))	11.00	3852.99	0.00
S(1)p(season)ψ(-1 + (state:tostate):season)	15.00	4043.87	0.00
S(1)p(1)ψ(-1 + (state:tostate):season)	14.00	4058.72	0.00
S(1)p(season)ψ(-1 + (state:tostate))	11.00	4434.56	0.00
S(1)p(1)ψ(-1 + (state:tostate))	10.00	4449.41	0.00

Table S1: **Model selection table.** The factors used to model survival (S), detectability (p) and transition probabilities (ψ) for each model are given in brackets. The + denotes additive affects, the * and : interactive effects. Further given are the number of parameters (npar), the Akaike information criterion, adjusted for small sample sizes (AICc), and the model weight.

model	npar	Δ AICc	weight
S(population size * state * season)p(population size * state * season)ψ(-1 + (state:tostate):season * population size)	49.00	0.00	0.99
S(population size * state * season)p(population size + state * season)ψ(-1 + (state:tostate):season * population size)	44.00	10.10	0.01
S(population size * state * season)p(state * season)ψ(-1 + (state:tostate):season * population size)	43.00	24.30	0.00
S(population size + state * season)p(population size * state * season)ψ(-1 + (state:tostate):season * population size)	44.00	89.95	0.00
S(population size + state * season)p(population size + state * season)ψ(-1 + (state:tostate):season * population size)	39.00	102.54	0.00
S(population size + state * season)p(state * season)ψ(-1 + (state:tostate):season * population size)	38.00	115.59	0.00
S(population size * state * season)p(population size * state * season)ψ(-1 + (state:tostate):season)	36.00	128.10	0.00
S(population size * state * season)p(population size + state * season)ψ(-1 + (state:tostate):season)	31.00	136.82	0.00
S(population size * state * season)p(state * season)ψ(-1 + (state:tostate):season)	30.00	149.23	0.00
S(state * season)p(population size * state * season)ψ(-1 + (state:tostate):season * population size)	43.00	172.63	0.00
S(state * season)p(population size + state * season)ψ(-1 + (state:tostate):season * population size)	38.00	184.75	0.00
S(state * season)p(state * season)ψ(-1 + (state:tostate):season * population size)	37.00	194.38	0.00
S(population size + state * season)p(population size * state * season)ψ(-1 + (state:tostate):season)	31.00	218.46	0.00
S(population size + state * season)p(population size + state * season)ψ(-1 + (state:tostate):season)	26.00	229.96	0.00
S(population size + state * season)p(state * season)ψ(-1 + (state:tostate):season)	25.00	241.27	0.00
S(state * season)p(population size * state * season)ψ(-1 + (state:tostate):season)	30.00	299.41	0.00
S(state * season)p(population size + state * season)ψ(-1 + (state:tostate):season)	25.00	310.34	0.00
S(state * season)p(state * season)ψ(-1 + (state:tostate):season)	24.00	318.45	0.00

Table S2: **Model selection table with continuous covariates**. The factors used to model survival (S), detectability (p) and transition probabilities (ψ) for each model are given in brackets. The + denotes additive affects, the * and : interactive effects. Further given are the number of parameters (npar), the Akaike information criterion, adjusted for small sample sizes (AICc), and the model weight.

Effect of density on state-dependent recapture probability in the breeding and off-breeding season

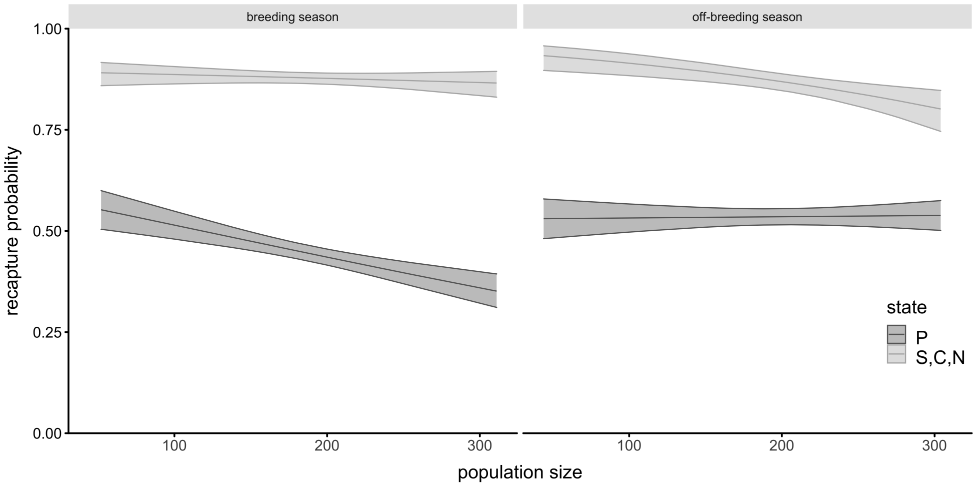

Figure S2: The effect of population size during the breeding (March to September) and the off-breeding season (October to February) on state-specific recapture probabilities in a free-living population of house mice (for the years 2007 to 2014). States are: P=prebreeder, second month or older, not yet breeding; S=solitary breeder, female raises a litter solitarily; C=communal breeder, female raises a litter communally; N=nonbreeder, female has no current litter but bred previously). Plotted are the estimates and 95% CI from a multi-state capture-mark-recapture model.

Effect of density on state-dependent survival and litter size in the off-breeding season

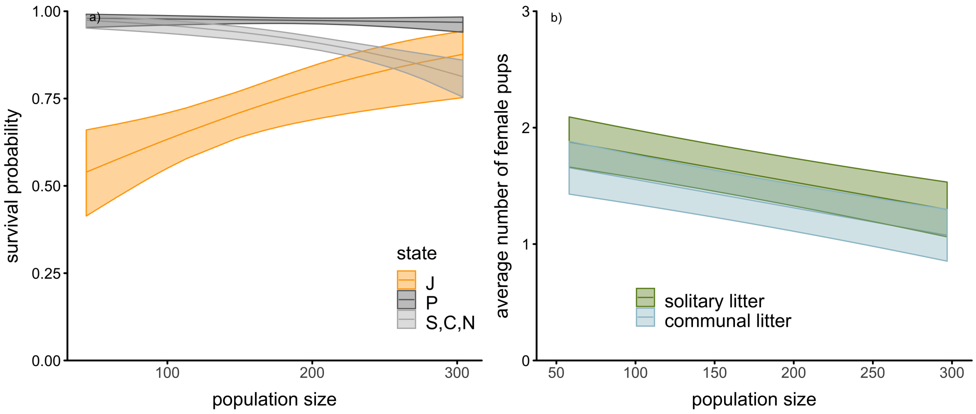

Figure S3: a) The effect of population size during the off-breeding season (October to February) on state-dependent survival (J=juvenile, first month of age; P=prebreeder, second month or older, not yet breeding; S=solitary breeder, female raises a litter solitarily; C=communal breeder, female raises a litter communally; N=nonbreeder, female has no current litter but bred previously) in a free-living population of house mice (for the years 2007 to 2014). Plotted are the estimates and 95% CI from a multi-state capture-mark-recapture model. b) Average number of female pups weaned for litters raised solitarily and communally during the off-breeding season.

Parameter estimates used for matrix population models

	overall		high density		low density	
	breeding season	off-breeding season	breeding season	off-breeding season	breeding season	off-breeding season
σ_J	0.40	0.71	0.49	0.80	0.28	0.60
σ_P	0.87	0.97	0.90	0.97	0.81	0.98
σ_S	0.93	0.90	0.93	0.89	0.95	0.97
σ_C	0.93	0.90	0.93	0.89	0.95	0.97
σ_N	0.93	0.90	0.93	0.89	0.95	0.97
$\psi_{J,P}$	1.00	1.00	1.00	1.00	1.00	1.00
b_P	0.16	0.04	0.14	0.04	0.23	0.05
b_S	0.35	0.00	0.22	0.00	0.52	0.00
b_C	0.33	0.00	0.24	0.00	0.59	0.00
b_N	0.29	0.05	0.31	0.03	0.26	0.08
c_P	0.73	0.80	0.79	0.82	0.58	0.74
c_S	0.46	0.00	0.63	0.00	0.36	0.00
c_C	0.65	0.00	0.77	0.00	0.51	0.00
c_N	0.72	0.53	0.74	0.64	0.65	0.40
f_S	1.72	1.65	1.54	1.48	1.89	1.83
f_C	1.42	1.35	1.32	1.26	1.67	1.61

Table S3: **Parameter estimates used to parameterize the matrix population models**. MSCMR-models were used to estimate the state- and season-dependent survival (σ_{state}), breeding probability conditional on survival (b_{state}) and the probability to breed communally conditional on breeding (c_{state}). The number of female pups per season and state was estimated using a linear model. The two seasons are the breeding (Mar-Sep) and off-breeding (Oct-Feb) season. Life-history states are abbreviated as follows: J=juvenile (first month of age), P=prebreeder (second month or older, not yet breeding), S=solitary breeder (female has a solitary litter), C=communal breeder (female has a communal litter), N=nonbreeder (female has no current litter, but bred previously).

Sensitivities of λ to changes in vital rate parameters

	overall breeding season			off-breeding season			low density breeding season			off-breeding season			high density breeding season			off-breeding season		
	sensitivity	lcl	ucl	sensitivity	lcl	ucl	sensitivity	lcl	ucl	sensitivity	lcl	ucl	sensitivity	lcl	ucl	sensitivity	lcl	ucl
σ_J	0.221	0.205	0.234	0.065	0.054	0.074	0.330	0.295	0.363	0.084	0.067	0.106	0.182	0.168	0.197	0.055	0.044	0.063
σ_P	0.349	0.331	0.366	0.794	0.738	0.843	0.280	0.252	0.304	0.538	0.462	0.619	0.389	0.367	0.411	0.850	0.794	0.899
σ_S	0.084	0.076	0.094	0.006	0.004	0.009	0.168	0.144	0.187	0.025	0.013	0.039	0.056	0.048	0.062	0.003	0.002	0.005
σ_C	0.179	0.167	0.190	0.016	0.011	0.020	0.197	0.173	0.221	0.032	0.022	0.042	0.173	0.161	0.188	0.011	0.007	0.016
σ_N	0.427	0.408	0.447	0.169	0.119	0.224	0.403	0.370	0.440	0.411	0.336	0.482	0.420	0.398	0.442	0.116	0.068	0.170
$\psi_{J,P}$	0.088	0.082	0.094	0.046	0.038	0.053	0.092	0.082	0.103	0.050	0.040	0.063	0.089	0.082	0.096	0.044	0.036	0.051
b_P	0.313	0.288	0.341	0.333	0.188	0.456	0.219	0.191	0.244	0.537	0.374	0.693	0.357	0.323	0.400	0.298	0.144	0.457
b_S	0.028	0.025	0.032	-0.006	-0.008	-0.003	0.053	0.044	0.066	-0.025	-0.038	-0.012	0.019	0.016	0.021	-0.003	-0.005	-0.001
b_C	0.058	0.053	0.062	-0.014	-0.018	-0.010	0.061	0.051	0.070	-0.031	-0.041	-0.021	0.056	0.052	0.062	-0.010	-0.015	-0.006
b_N	0.138	0.126	0.151	0.291	0.243	0.341	0.139	0.115	0.167	0.300	0.228	0.436	0.134	0.122	0.151	0.290	0.215	0.373
c_P	-0.005	-0.008	-0.003	-0.007	-0.009	-0.005	-0.001	-0.004	0.002	-0.003	-0.005	-0.002	-0.004	-0.006	-0.001	-0.006	-0.008	-0.003
c_S	-0.002	-0.003	-0.001	0.000	0.000	0.000	-0.001	-0.004	0.002	0.000	0.000	0.000	-0.001	-0.001	-0.000	0.000	0.000	0.000
c_C	-0.004	-0.006	-0.002	0.000	0.000	0.000	-0.001	-0.005	0.003	0.000	0.000	0.000	-0.002	-0.003	-0.001	0.000	0.000	0.000
c_N	-0.009	-0.013	-0.005	-0.003	-0.004	-0.002	-0.001	-0.005	0.003	-0.003	-0.005	-0.002	-0.006	-0.010	-0.002	-0.002	-0.003	-0.001
f_3	0.018	0.016	0.021	0.009	0.007	0.012	0.024	0.020	0.028	0.013	0.009	0.019	0.016	0.014	0.018	0.008	0.005	0.010
f_3	0.039	0.037	0.042	0.023	0.019	0.026	0.028	0.023	0.031	0.017	0.012	0.024	0.049	0.044	0.053	0.026	0.021	0.031

Table S4: **Sensitivities and bootstrapped 95% CI of λ to changes in vital rate parameters in season- and density-dependent matrix population models.** Vital rates are life-history state-dependent survival (σ_{state}), transition probability from J to P ($\psi_{J,P}$), breeding probability conditional on survival (b_{state}), probability to breed communally conditional on breeding (c_{state}) and the number of female pups produced on average (f_{state}). Life-history states are abbreviated as follows: J=juvenile (first month of age), P=prebreeder (second month or older, not yet breeding), S=solitary breeder (female has a solitary litter), C=communal breeder (female has a communal litter), N=nonbreeder (female has no current litter, but bred previously).

Model selection tables for the effect of female condition

(Intercept)	pop. size	mass	state	pop. size:mass	pop. size:state	mass:state	pop. size:mass:state	df	AICc	Δ AICc	weight
A) probability to breed											
-0.51	-1.39	-0.48	+		+	+		13	1293.5	0.0	0.63
-0.51	-1.43	-0.46	+	0.19	+	+		14	1295.3	1.8	0.26
-0.48	-1.64	-0.41	+	1.06	+	+	+	17	1296.9	3.4	0.11
-0.74	-1.21	0.51	+		+			10	1314.9	21.4	0.00
-0.74	-1.22	0.51	+	0.04	+			11	1316.9	23.5	0.00
-0.48		-0.06	+			+		9	1317.5	24.1	0.00
-0.48	0.15	-0.01	+			+		10	1318.4	24.9	0.00
-0.46	0.11	-0.00	+	0.32		+		11	1319.4	26.0	0.00
-0.62	-1.29		+		+			9	1321.9	28.4	0.00
-0.63	0.15	0.54						4	1333.6	40.2	0.00
-0.64	0.13	0.55		0.23				5	1335.1	41.6	0.00
-0.61		0.50	+					6	1338.2	44.7	0.00
B) probability to breed communally											
1.11	1.18	-0.74		-1.61				5	430.88	0.00	0.68
1.99	2.85	-0.72	+	-1.42	+			11	434.39	3.50	0.12
1.26	1.12	-0.75	+	-1.56				8	434.98	4.09	0.09
2.25	2.97	-1.56	+	-1.72	+	+		14	437.15	6.26	0.03
1.43	1.14	-1.42	+	-1.77		+		11	438.50	7.61	0.02
1.00	0.82	-0.49						4	438.55	7.67	0.01
1.79	2.47		+		+			9	438.67	7.79	0.01
1.93	2.51	-0.45	+		+			10	438.72	7.84	0.01
0.91	0.79							3	439.66	8.78	0.01
1.20	0.79	-0.46	+					7	442.11	11.23	0.00
1.08	0.78		+					6	442.37	11.49	0.00
1.98	2.49	-0.68	+		+	+		13	443.58	12.70	0.00

Table S5: Model (GLMMs) selection tables for (A) the probability to breed, and conditional on breeding, (B) the probability to breed communally in the next month. Continuous covariates were standardised. Factors included are defined as follows: pop.size: the number of adults in the population, mass: female body mass, state: female life-history state (prebreeder, solitary breeder, communal breeder, nonbreeder). Abbreviations: df: degrees of freedom, AICc: Akaike information criterion, adjusted for small sample sizes, weight: relative model weights.

Female body mass

We tested the effect of population density and a female's life-history state on her body mass [g] using a linear mixed model. Female identity was used as a random factor to account for multiple weight measures per female. The full model containing both density (the number of adults in the barn, standardised), female state and their interaction had a lower AICc than models containing only an additive effect, or only one of the factors. Population density led to a slight increase in female body mass for solitarily breeding females and similarly a slight decrease for communally breeding females. Such patterns are expected if, as we hypothesize in the main text, the increased competition at higher densities resulted in only the very best (*i.e.* heaviest) females being able to raise their litters solitarily.

	estimate	SE
(Intercept)	25.62	0.25
density	-1.05	0.51
state N	0.98	0.29
state P	-3.85	0.28
state S	0.65	0.47
density:state N	0.90	0.58
density:state P	1.29	0.58
density:state S	2.80	0.99

Table S6: Model estimates from a linear mixed model testing the effect of female life-history state and population density on female body mass. Female identity was used as a random factor. The intercept represents a communally breeding female (C) at the average population density. Density was measured as the number of adult mice in the barn and was standardised for the analysis. Life-history states are abbreviated as follows: P=prebreeder (second month or older, not yet breeding), S=solitary breeder (female has a solitary litter), C=communal breeder (female has a communal litter), N=nonbreeder (female has no current litter, but bred previously).

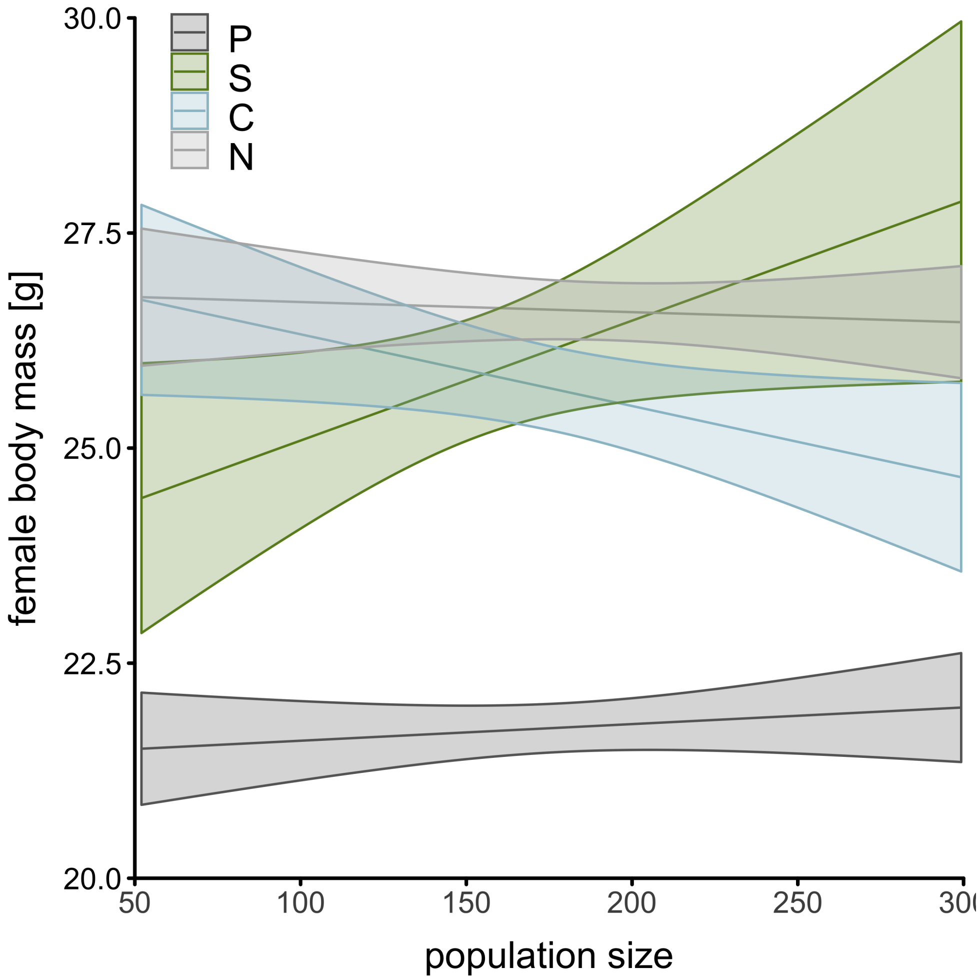

Figure S4: The effect of population density and a female's state on her body mass. Plotted are model mean estimates and 95% CI from a linear mixed model. The states are: P=prebreeder, S=solitarily breeding female, C=communally breeding female and N=nonbreeder.
