# Supplementary material for: Cooperation by necessity: condition- and density-dependent reproductive tactics of female house mice
Source: Commun Biol. 2022 Apr 12;5:348. doi: 10.1038/s42003-022-03267-2 (PMC9005510; doi:10.1038/s42003-022-03267-2)
Supplement: Supplementary file 3 — Description of Additional Supplementary Files [file 42003_2022_3267_MOESM3_ESM.pdf]

Description of Additional Supplementary Files

File name: Supplementary Data 1:
Description: Encounter histories

File name: Supplementary Data 2:
Description: female condition

File name: Supplementary Data 3:
Description: litters

File name: Supplementary Data 4:
Description: covariates
