# Supplementary material for: Cooperation by necessity: condition- and density-dependent reproductive tactics of female house mice
Source: Commun Biol. 2022 Apr 12;5:348. doi: 10.1038/s42003-022-03267-2 (PMC9005510; doi:10.1038/s42003-022-03267-2)
Supplement: Supplementary file 8 — Reporting Summary [file 42003_2022_3267_MOESM8_ESM.pdf]

nature research

Corresponding author(s): Manuela Ferrari

Last updated by author(s): Feb 10, 2022

# Reporting Summary

Nature Research wishes to improve the reproducibility of the work that we publish. This form provides structure for consistency and transparency in reporting. For further information on Nature Research policies, see Authors & Referees and the Editorial Policy Checklist.

## Statistics

For all statistical analyses, confirm that the following items are present in the figure legend, table legend, main text, or Methods section.

| n/a | Confirmed | |
|---|---|---|
| ☐ | ☒ | The exact sample size (*n*) for each experimental group/condition, given as a discrete number and unit of measurement |
| ☐ | ☒ | A statement on whether measurements were taken from distinct samples or whether the same sample was measured repeatedly |
| ☐ | ☒ | The statistical test(s) used AND whether they are one- or two-sided<br>*Only common tests should be described solely by name; describe more complex techniques in the Methods section.* |
| ☐ | ☒ | A description of all covariates tested |
| ☐ | ☒ | A description of any assumptions or corrections, such as tests of normality and adjustment for multiple comparisons |
| ☐ | ☒ | A full description of the statistical parameters including central tendency (e.g. means) or other basic estimates (e.g. regression coefficient) AND variation (e.g. standard deviation) or associated estimates of uncertainty (e.g. confidence intervals) |
| ☒ | ☐ | For null hypothesis testing, the test statistic (e.g. *F*, *t*, *r*) with confidence intervals, effect sizes, degrees of freedom and *P* value noted<br>*Give P values as exact values whenever suitable.* |
| ☒ | ☐ | For Bayesian analysis, information on the choice of priors and Markov chain Monte Carlo settings |
| ☒ | ☐ | For hierarchical and complex designs, identification of the appropriate level for tests and full reporting of outcomes |
| ☒ | ☐ | Estimates of effect sizes (e.g. Cohen's *d*, Pearson's *r*), indicating how they were calculated |

*Our web collection on statistics for biologists contains articles on many of the points above.*

## Software and code

Policy information about availability of computer code

| | |
|---|---|
| Data collection | No special computer code was used to collect the data. |
| Data analysis | All analyses were conducted with R and MARK (througout the RMark interface). All used packages and the versions are given in the method section. |

For manuscripts utilizing custom algorithms or software that are central to the research but not yet described in published literature, software must be made available to editors/reviewers. We strongly encourage code deposition in a community repository (e.g. GitHub). See the Nature Research guidelines for submitting code & software for further information.

## Data

Policy information about availability of data

All manuscripts must include a data availability statement. This statement should provide the following information, where applicable:

- Accession codes, unique identifiers, or web links for publicly available datasets
- A list of figures that have associated raw data
- A description of any restrictions on data availability

All data analysed during this study are included in the article (and its supplementary materials).

# Field-specific reporting

Please select the one below that is the best fit for your research. If you are not sure, read the appropriate sections before making your selection.

☐ Life sciences ☐ Behavioural & social sciences ☒ Ecological, evolutionary & environmental sciences

# Ecological, evolutionary & environmental sciences study design

All studies must disclose on these points even when the disclosure is negative.

| | |
|---|---|
| Study description | We estimated life-history parameters in a population of free living house mice, trying to asses the effect of two different breeding tactics on the overall population growth rate. We further analysed the effect of density and female body mass and on her likelihood to use one or the other breeding tactic. |
| Research sample | All females that were present in the study population between 2007 and 2014 and for which all necessary information was available (date of birth, breeding tactic they used for each litter...). |
| Sampling strategy | Sample size was limited by the available females in the population and we therefore had no way to influence it. However, given the duration of the study, a large number of females could be included (4003 females). |
| Data collection | All captured adults were injected with a RFID tag, when they were ≥ 17.5g and a ear punch (genetic tissue sample) was taken for parentage analyses. Pups found were counted, weighted and a tissue sample taken when they were around 13 days old. Furthermore it was recorded whether they were alone in the nest (solitary litter), or together with one or several other litters (communal litter). Genetic information (from the tissue samples) was used to determine whether pups belonged to the same, or different litters. |
| Timing and spatial scale | Data used in this study was collected continuously between January 2007 and December 2014 in a single population of free-living house mice. |
| Data exclusions | If pups were only sampled as sub-adults and we therefore had no information about the way they were raised (solitarily or communally), they were not included as breeding event, the same was true for pups that could not be assigned to a mother with a certainty of 95%. |
| Reproducibility | The study was based on observational data from a single free-living population over several years and it was therefore not possible to repeat it, which would have required an additional population. |
| Randomization | The study was based on observational data from a free-living population and no experiments were conducted, that would have required randomization. |
| Blinding | Again, given the nature of the study (observational data from a free-living population over several years), this was not an issue. |

Did the study involve field work? ☐ Yes ☒ No

# Reporting for specific materials, systems and methods

We require information from authors about some types of materials, experimental systems and methods used in many studies. Here, indicate whether each material, system or method listed is relevant to your study. If you are not sure if a list item applies to your research, read the appropriate section before selecting a response.

## Materials & experimental systems

| n/a | Involved in the study | |
|---|---|---|
| ☒ | ☐ | Antibodies |
| ☒ | ☐ | Eukaryotic cell lines |
| ☒ | ☐ | Palaeontology |
| ☐ | ☒ | Animals and other organisms |
| ☒ | ☐ | Human research participants |
| ☒ | ☐ | Clinical data |

## Methods

| n/a | Involved in the study | |
|---|---|---|
| ☒ | ☐ | ChIP-seq |
| ☒ | ☐ | Flow cytometry |
| ☒ | ☐ | MRI-based neuroimaging |

## Animals and other organisms

Policy information about studies involving animals; ARRIVE guidelines recommended for reporting animal research

| | |
|---|---|
| Laboratory animals | No laboratory animals were used in this study. |
| Wild animals | The data was collected from a intensively monitored population of free living Eastern European House mice (Mus musculus domesticus). The population lives in a former agricultural building and the house mice are provided with food, water, nesting material and shelters for breeding (artificial nest boxes) all year round. The building is protected against larger predators, but the house mice and other small mammals can enter and leave freely. In this study 4003 females were studied, as well as their corresponding litters. |
| Field-collected samples | Animals were not removed from their population for this study. |

| | |
|---|---|
| Ethics oversight | The data collection and all methods used for the presented study were approved by the Swiss Veterinary office (Canton Zurich, license numbers 215/2006 and 51/2010) |

Note that full information on the approval of the study protocol must also be provided in the manuscript.
